# Transposon insertion sequencing reveals novel hypermutator genes in *Acinetobacter baumannii*

**DOI:** 10.1128/mbio.00966-25

**Published:** 2025-06-27

**Authors:** Francesca L. Short, Ram Maharjan, Liping Li, Nusrat Afrin, Natasha Delgado, Christine J. Boinett, Julian Parkhill, Amy K. Cain, Ian T. Paulsen

**Affiliations:** 1 Department of Microbiology, Biomedicine Discovery Institute, Monash University 214149, Clayton, Victoria, Australia; 2 ARC Centre of Excellence in Synthetic Biology, School of Natural Sciences, Macquarie University 7788, Sydney, New South Wales, Australia; 3 Wellcome Sanger Institute 47665, Hinxton, United Kingdom; Vanderbilt University School of Medicine, Nashville, Tennessee, USA

**Keywords:** hypermutator, tn-seq, tradis, mutation rate, *Acinetobacter*

## Abstract

Mutation rates in bacteria are an important determinant of adaptation to new environments and success in different niches. In some bacterial pathogens, “hypermutator” variants—most often associated with mutations in components of the DNA mismatch repair system—are associated with increased antibiotic resistance and poorer patient outcomes. We report the serendipitous finding of novel hypermutator genes in *Acinetobacter baumannii* through genome-scale mutant fitness screening. Exposure of a transposon insertion mutant library of *A. baumannii* to extended weak antibiotic selection resulted in selection for mutations that directly increased fitness as expected, but also revealed genes where transposon insertion indirectly increased fitness due to elevated general mutation rates. Three novel hypermutator genes were confirmed in *A. baumannii: nusB*, encoding a transcription antiterminator; ABUW_0208, encoding a hypothetical protein; and ABUW_2121, which encodes a sulfite transporter. We find selection for hypermutator variants in transposon insertion sequencing (TIS) data sets from diverse bacteria under various antibiotic treatments. Our results expand the range of biological functions linked to hypermutator phenotypes in bacteria and provide a workflow for the identification of putative hypermutators by TIS.

**IMPORTANCE:**

All organisms have the capacity for evolution through mutation. Bacteria with high mutation rates have a survival advantage in some stressful environments because they generate beneficial mutations more frequently. “Hypermutators” are bacterial strains that carry gene inactivations that increase general mutation rates. These variants are important in chronic infections, as their increased genetic diversity allows higher drug resistance and prolonged survival in the host. Only a few different hypermutator genes are known, and there is no high-throughput method for their identification. We have made the serendipitous finding that hypermutator genes can be identified by genome-wide mutant fitness screening under specific selection conditions. We have identified novel hypermutator alleles in the notorious hospital pathogen *Acinetobacter baumannii* and show that hypermutator variants can be detected in screens of a wide range of pathogens.

## OBSERVATION

Bacterial evolution ultimately depends on the generation of genetic diversity through mutation. Though the majority of mutations are deleterious or fitness-neutral, in some scenarios, elevated mutation rates can be beneficial, increasing the rate of adaptation (1–3). Mutation rates in bacteria can vary, either because they are plastic and can be fine-tuned depending on the environment, or because mutations that genetically increase the mutation rate of a genome can increase the adaptation rate in stressful environments (4–8). Bacterial isolates with stably increased genome-wide mutation rates are termed hypermutators (9). Such strains have an enhanced ability to evolve antibiotic resistance and often arise during chronic infections (10–14). Mutators with as little as a 10-fold increase in mutation rates can show accelerated evolution of antibiotic resistance (15). To date, mutations in several well-conserved DNA repair genes (e.g., *mutS*, *mutL,* and *mutT*) have been identified as resulting in a hypermutator phenotype (4, 16), and mutation rate impacts for additional genes in *Escherichia coli* have been identified through evolution experiments and forward genetic screens (14, 17). Though the importance of hypermutation is well-established both *in vivo* and *in vitro*, we have an incomplete understanding of the genetic causes of mutator phenotypes, particularly outside of the model organism *E. coli*.

*Acinetobacter baumannii* is an “ESKAPE” group opportunistic human pathogen for which new antibiotics are urgently needed (18). One of the few therapeutic options for carbapenem-resistant *A. baumannii* is tigecycline, a ribosome-targeting antibiotic (19, 20). Resistance to tigecycline develops through overexpression of certain efflux pumps, target-site mutations, or mobile resistance genes (21–24). Tigecycline-resistant *A. baumannii* mutants arise frequently during therapy (25–27), and some mutants are also hypermutators (27, 28).

Here, we make the unexpected discovery that hypermutator genes can be identified through transposon insertion sequencing (TIS) after extended, low-level antibiotic exposure, resulting in weak selection for mutations that increase fitness under the selection conditions of the experiment. TIS is a high-throughput genetic fitness profiling technique involving saturation random transposon mutagenesis combined with selection and sequencing (29). We leverage this approach to discover several novel hypermutator genes in *A. baumannii* and show that hypermutators are selected in a wide range of TIS screens of different bacteria and antibiotics.

**Fig 1:**
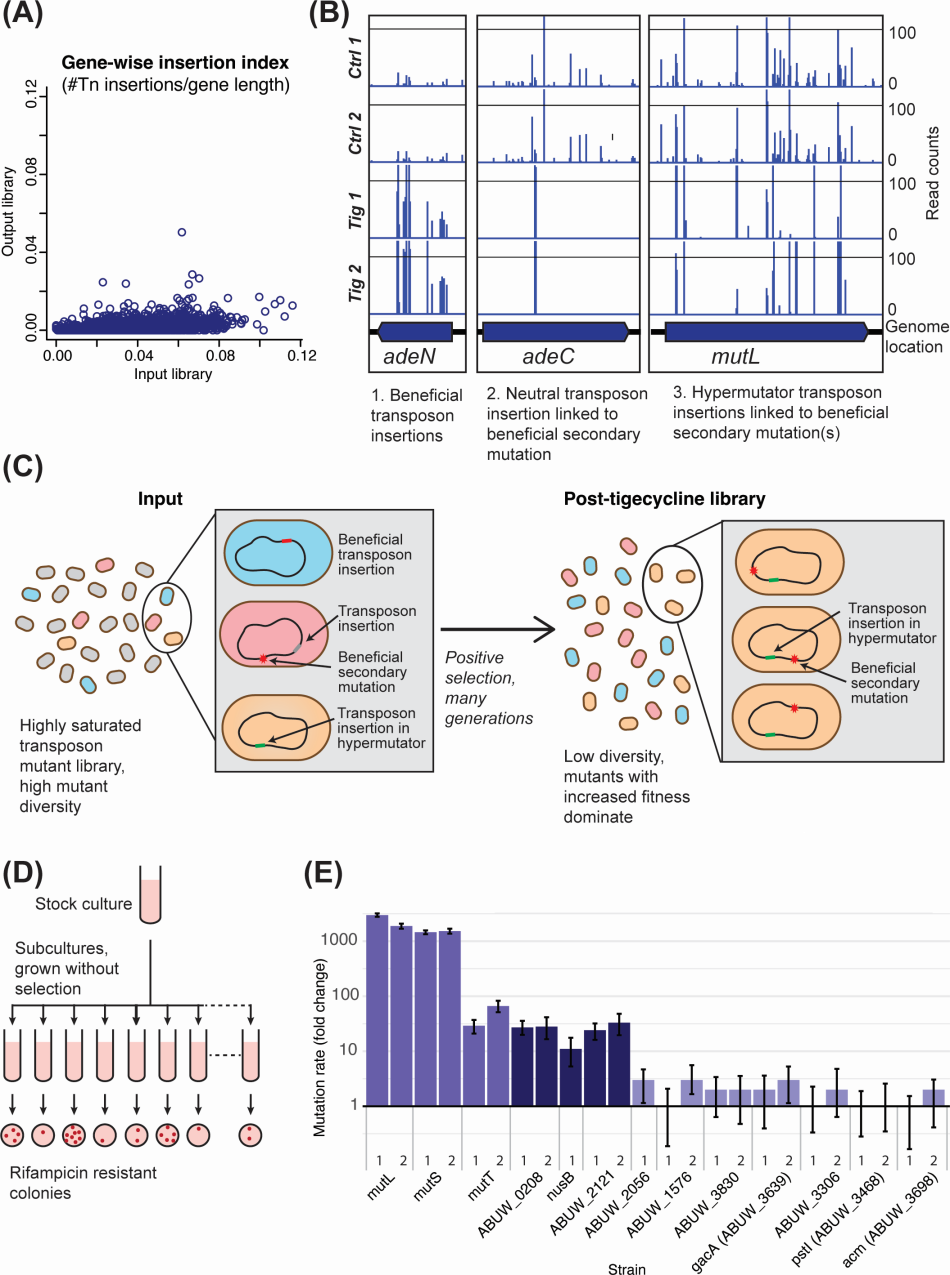
Schematic of experiment design and selection. (**A**) Genewise transposon insertion density before (*x* axis) and after (*y* axis) weak antibiotic selection. The majority of genes showed a dramatic reduction in insertion index following the experiment. (**B**) Raw transposon insertion sequencing plots for three genes showing different selection profiles. Vertical blue line locations indicate transposon insertion sites, while line height indicates read count at each site. If transposon insertion within a gene increases fitness directly, all transposon mutations within a gene will be selected, and this will be reproducible between replicates (e.g., *adeN*). In contrast, reproducible selection of just a single transposon mutant within a gene (e.g., *adeC*) is likely to be caused by a rare beneficial secondary mutation linked to the transposon insertion. Transposon insertions that increase general mutation rates (e.g., *mutL*) are positively selected because they facilitate fitness-increasing linked secondary mutations; however, the selection is not consistent between replicates. (**C**) Schematic presentation of input and post-antibiotics treated library. The library is highly saturated (>100,000 unique transposon insertions) so that every gene is independently mutated multiple times. The majority of transposon insertions are fitness-neutral (grey cells) and will be depleted in a positive selection experiment; however, a small proportion will increase fitness (blue cells). Neutral transposon insertions can “hitchhike” with beneficial secondary mutations that occur by chance in the same cell (pink cells) and will also increase in abundance following positive selection. Finally, if a transposon insertion causes a hypermutator phenotype (orange cells), this may be indirectly selected due to it facilitating beneficial mutations elsewhere in the genome during selection. The post-selection library is comprised almost exclusively of mutants with increased fitness, which can be a consequence of the transposon insertion itself, or a beneficial linked secondary mutation with or without a hypermutator phenotype. (**D**) Schematic of Luria-Delbrück fluctuation test to examine mutation rates in candidate hypermutator genes. (**E**) Mutation rates for *Acinetobacter baumannii* AB5075 and its Tn*26* insertion mutants, expressed as fold change relative to wild type with 95% confidence intervals. Mutation rates are based on rifampicin resistance (Rif^R^) mutations from rifampicin sensitive (Rif^S^) as determined by the fluctuation test with 30 independent cultures. The *mutL*, *mutS,* and *mutT* mutants were expected to have elevated mutation rates, while ABUW_1576 and ABUW_3698 were not. For the majority of genes of interest, more than one transposon insertion mutant was tested; precise mutant identities are provided in Table S1. Raw mutation rates and confidence intervals are provided in Table S1.

The aim of this work was to identify *A. baumannii* genes contributing to survival in the presence of tigecycline. We exposed a saturated *A. baumannii* BAL062 transposon insertion library (30) to tigecycline at 0.25× MIC for 16 hours (20 generations). Unexpectedly, the transposon library diversity collapsed from 128,000 unique transposon insertion sites (UIS) to ~7,000 UIS following exposure, with most genes showing a dramatic decrease in transposon insertion density (Fig. 1A). Without antibiotic treatment, the density of transposon insertions per gene followed a characteristic bimodal distribution with clear separation of essential and non-essential genes, whereas following tigecycline selection, most genes contained very few insertions (Fig. S1A). As a population bottleneck in this *in vitro* experiment was implausible, we reasoned that the collapse in diversity occurred because transposon mutants with increased fitness in the presence of tigecycline had proliferated, overwhelming cells with unchanged fitness.

### Mutants selected during low-level tigecycline exposure

We initially identified 408 genes with mutants showing positive selection (log2 fold change > 3, corrected *P*-value < 0.1; Table S1). The dominant hit was *adeN*; all transposon insertions across the length of the gene showed dramatically increased reads in both replicates (Fig. 1B), with excellent correlation in reads per transposon site (Fig. S1B). AdeN represses transcription of the AdeIJK efflux pump. Mutation of *adeN* relieves this repression, increasing resistance to several drugs including tigecycline in clinical MDR isolates (31, 32).

The remaining positively selected mutants showed two different profiles: (i) selection of a single transposon insertion within a gene, consistent between replicates (e.g., *adeC*, Fig. 1B), and (ii) selection of several different transposon insertions within the gene, but with low consistency between replicates (e.g., *mutL*, Fig. 1B; Fig. S1B). The first profile is presumed to arise from the presence of an adaptive secondary mutation, unrelated to the transposon insertion, as has previously been noted in positive-selection TIS data (33, 34). Genes displaying the second profile included the known hypermutators *mutL* and *mutS*. We therefore hypothesized that our conditions had indirectly selected for bacteria with higher mutation rates; such cells increase in frequency under stressful conditions, as mutator alleles can hitchhike along with the beneficial mutations they trigger, in this case during the 16 hours of growth in the presence of tigecycline (15). A schematic of the experiment and the population after selection is shown (Fig. 1C). The initial transposon mutant pool includes many thousands of unique transposon insertions, as well as some level of transposon-independent genetic variation (spontaneous mutations). The final mutant pool includes cells where a transposon mutation increases fitness directly (e.g., *adeN*), cells with a pre-existing beneficial secondary mutation linked to a single neutral transposon insertion (e.g., *adeC*), and cells that have gained an adaptive secondary mutation during the experiment due to the presence of a transposon insertion that increases general mutation rates (e.g., *mutL* transposon insertions). Note that as *A. baumannii* is naturally competent (35), there is a possibility of external DNA acquisition and incorporation during the experiment, though we consider this unlikely because the growth conditions used are not known to promote natural competence. Our experiment therefore presents the opportunity to identify mutator alleles in *A. baumannii*.

### Identification of novel hypermutator genes in *A. baumannii*

To identify putative hypermutators, we examined the data for genes showing similar selection profiles to the known hypermutator genes *mutL*, *mutS,* and *mutT* (Fig. 1B; Fig. S2). The methyl-directed mismatch repair proteins encoded by *mutL* and *mutS* are responsible for the repair of base-base mismatches and small nucleotide insertion/deletions (9), while *mutT* encodes an enzyme that hydrolyzes a mutagenic oxidized form of dGTP to the monophosphate form (36). Inactivation of any of these genes significantly increases spontaneous mutation rates in bacteria (9, 16, 36), and all three genes had high mutant abundance yet uneven selection of independent transposon insertions in the tigecycline selection experiment, suggesting these transposon insertions hitchhiked with a linked beneficial mutation. Note that the nature of any tigecycline resistance mutations linked to the hypermutators is not known, although these may include base substitution mutations in the *rpsJ* gene, which leads to tigecycline resistance in some bacteria including *A. baumannii* (28, 37).

Putative novel hypermutators were identified as follows: first, genes were filtered for those showing positive selection of mutants, with essential or near-essential genes excluded (cutoffs log2FC > 1.0 and logCPM ≥ 4.0). Then, genes were selected in which the transposon insertion density observed after the experiment (expressed as insertion index, #insertions/gene length) was at least 0.25. Genes with fewer than four unique insertion sites in the control condition were also excluded, to allow evaluation of mutant retention and comparison of this between replicates (Fig. S1). Finally, raw transposon insertion plots were manually examined for each putative hit to confirm the uneven selection of insertion sites across the gene (Fig. S2). While the correlation of read counts at each individual transposon insertion site was also calculated (Table S1), this was not applied as an explicit filtering criterion. Overall, 24 genes met the criteria for potential hypermutators, including genes with metabolic, regulatory, and hypothetical functions, along with *mutL*, *mutS,* and *mutT* (Table S1).

We investigated a selection of these putative hypermutators in depth using the fluctuation test for rates of spontaneous rifampicin-resistance (Fig. 1D), which is usually conferred by base-pair substitutions in the *rpoB* gene (38). Mutation rates were measured in eight *A. baumannii* AB5075 candidate hypermutators, as well as two genes with positive selection but a consistent profile between replicates. Inactivation of *mutL, mutS,* and *mutT* in *A. baumannii* AB5075_UW increased mutation rates by 1,453-, 2,977-, and 30-fold, respectively, compared to WT (Fig. 1E; Table S1), consistent with their role in DNA repair (36). Mutation rates in ABUW_0208 (hypothetical protein) and ABUW_2121 (predicted sulfite exporter) mutants were 0.26 × 10^−8^ (95% CI, 0.19-0.34 × 10^−8^) and 0.23 × 10^−8^ (95% CI, 0.15-0.31 × 10^−8^) mutations per generation, respectively, which is 27- and 24-fold higher than the WT and comparable with *mutT* inactivation. Similarly, the transcription antiterminator, *nusB*, showed an 11-fold higher mutation rate. Mutations in three genes (ABUW_2056, ABUW_1576, and ABUW_3830) caused a two- to three-fold increase in mutation rate, which was not statistically significant, while disruption of *gacA*, ABUW_3468, ABUW_3306, and ABUW_3698 had no impact on mutagenesis. In summary, from the initial pool of eight candidates, we identified three novel hypermutator genes, exhibiting mutation frequencies similar to the well-known hypermutator *mutT*. However, a limitation of these experiments is that certain mutation types, like indels, may go undetected. This is because the rifampicin resistance phenotype relies primarily on base-pair substitutions in an essential gene, *rpoB* (38). Note that of the five candidate hypermutators that did not show elevated mutation rates, four had comparatively low signals of positive selection (log2FC < 3), suggesting a more stringent threshold would be appropriate in the future; in addition, no hypermutator phenotypes were identified in genes with a read count per insertion site correlation of >0.35 (Table S1).

The mechanism underpinning these hypermutator phenotypes is unknown. It is possible that the loss of *nusB* (transcription antiterminator) could detrimentally alter nucleotide availability and cycling, impacting DNA replication and repair. Further research is required to determine the mechanism by which the hypothetical protein ABUW_0208 and the sulfite exporter ABUW_2121 impact mutation rates. Overall, our results identify novel hypermutator genes in *A. baumannii* through TIS and suggest a broad range of cellular functions are important for maintaining DNA replication and repair fidelity.

### Hypermutator detection in TIS data from diverse bacteria and selection conditions

**Fig 2:**
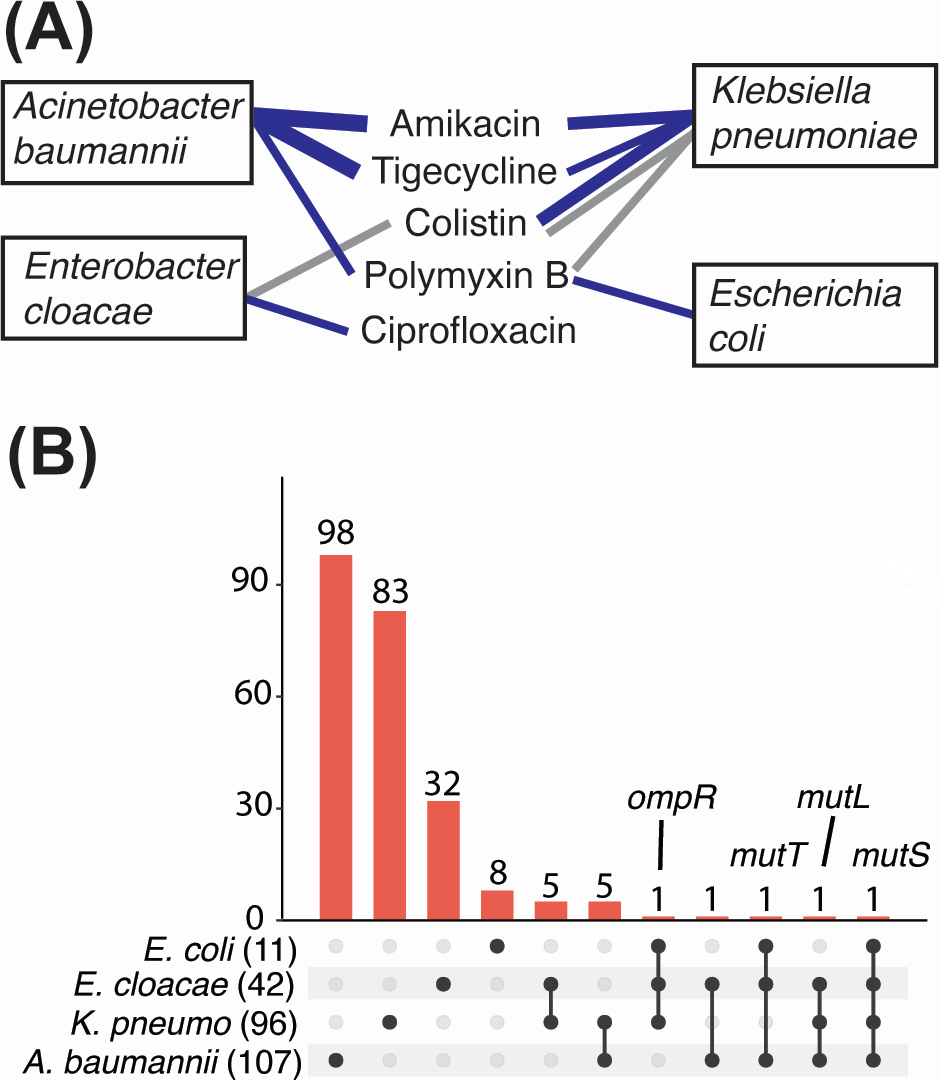
(**A**) Overview of multispecies TraDIS analysis for hypermutator signatures. Eleven screens (including the initial *A. baumannii* tigecycline experiment) showed a collapse in library diversity following selection; eight of these showed the selection of *mutS*, *mutL,* or both (blue lines), while three did not (grey lines). Line thickness corresponds to the number of positively selected genes identified in each screen. (**B**) Upset plot showing the intersection between hypermutator candidates identified in four species, with key genes indicated. Only genes where homologs were identified in one or more of the other species are included. Full details are provided in Table S2.

We then wished to determine whether the selection of hypermutator alleles is common in TIS experiments. We examined data from our previous large-scale functional genomics projects, which addressed five antibiotics in diverse gram-negative ESKAPE pathogens (ENA study accession PRJEB3226). We identified 10 experiments that showed a collapse in library diversity similar to our *A. baumannii* tigecycline exposure experiment (Fig. 2A). These data sets were analyzed as before, and genes showing positive selection and high insertion site retention were examined (see Materials and Methods). Eight distinct screens encompassing four species: *E. coli*, *Enterobacter cloacae*, *Klebsiella pneumoniae*, and *A. baumannii*, and five antibiotic treatments: polymyxin B, tigecycline, colistin, ciprofloxacin, and amikacin showed positive selection of *mutS*, *mutL,* or both (Fig. 2; Table S2). The three screens that did not show selection of hypermutators employed colistin or polymyxin B as the antibiotic. The intersection of the hypermutator candidates among the four species was examined; of 402 genes across the four species and antibiotic treatments, homologues in one or more of the other species could be identified for 255. Sixteen of these genes were identified as putative hypermutators in more than one species including *mutS*, *mutL,* and *mutT* (Fig. 2B; Table S2). Selection of hypermutators in TIS experiments is therefore a common outcome, and designing experiments specifically for this purpose is likely to be feasible if known hypermutator strains are available for optimization of selection conditions.

### Conclusions

We have identified and validated three novel hypermutator genes in *A. baumannii*, using an antibiotic selection TIS screen. The varied functions of our newly identified hypermutators indicate that wide-ranging cellular activities are important for maintaining low mutation rates, beyond the known mutator alleles affecting nucleotide processing and DNA mismatch repair. Our study shows how two factors that are normally compromising in TIS experiments—secondary mutations and proliferation of fast-growing mutants—can facilitate the discovery of new biological activities. In light of increasing evidence that bacteria fine-tune their mutation rates for evolutionary success, we suggest that the identification of hypermutators by TIS could be a productive approach for future research.

### Bacterial TraDIS and data analysis

Mutant libraries in *K. pneumoniae* (39), *E. cloacae* (40), *E. coli* (41), and *A. baumannii* (30) were grown in Mueller-Hinton II broth for 16 hours at 37°C. Antibiotics were added at 1/4 MIC (tigecycline and ciprofloxacin) or 1/10 MIC (amikacin, colistin, and polymyxin B). All experiments were conducted in duplicate, with a control of growth without added antibiotic. Genomic DNA was extracted, sequenced, and analyzed according to the previously published protocols in the BioTraDIS pipeline (42). The following thresholds were applied to identify putative hypermutator genes in the initial *A. baumannii* tigecycline selection data: (i) log2FC ≥ 1 following antibiotic selection, (ii) logCPM ≥ 3, (iii) unique insertion sites in the input library ≥ 3, and (iv) an insertion index ratio (indicating the proportion of starting transposon mutations recovered in the output library) of ≥0.25. Criterion 4 was chosen empirically based on the profile of *mutT_2*. Manual examination of the mapped reads was conducted using the Artemis genome browser, and correlation between read counts at individual insertion sites within each gene for the two replicates was determined in R using a linear model on log-transformed non-zero read counts.

Analysis across different species and antibiotic treatments was performed using the same criteria as for *A. baumannii* tigecycline, except the insertion index ratio threshold was set as the top 5% within the experiment; exact values are provided in Table S2. A BLAST database was constructed from all protein sequences from the four species, and translated candidate hypermutator gene sequences from all screens where either *mutS* or *mutL* were positively selected were used to query this database. Putative homologs identified by BLASTp were filtered for >25% sequence identity and >70% query coverage, and the top hit (lowest e value) within each genome was retained. The upset plot was generated using Intervene (43).

### Mutation rate assay

Resistance to rifampicin (Rif^R^) in *A. baumannii* strain AB5075_UW (rifampicin sensitive, Rif^S^) and its Tn26 insertion mutants (44) was measured to determine mutation rates (Rif^S^ → Rif^R^ assay). A single colony of each strain was inoculated in Luria Bertani (LB) broth and allowed to propagate overnight at 37°C with shaking at 200 rpm. The overnight cultures were diluted in fresh LB medium and allowed to grow to an OD_600nm_ of 0.6. The exponential cultures were diluted 10,000-fold, and 150 µL distributed into 96-well plates (30 wells per strain) and incubated at 37°C with shaking at 200 rpm. Aliquots (100 µL) were plated on LB agar supplemented with 25 µg/ml rifampicin (Sigma-Aldrich), and incubated for 24 h at 37°C to detect Rif^R^ mutant colonies. For total colony-forming unit (CFU) counts, aliquots of appropriately diluted cultures were plated on LB agar plates. The mutation rates were calculated from the total and Rif^R^ CFU counts per culture using the Luria-Delbrück fluctuation test (45), Ma-Sandri-Sarkar maximum likelihood analysis (46), and fluctuation analysis calculator (47).

## Data Availability

Raw sequencing data are available through NCBI/ENA/DDBJ under study PRJEB3226. Individual sample accessions are provided in Table S2.

## SUPPLEMENTAL MATERIAL

Supplemental material mbio.00966-25-s0001.docx Supplemental figures and table captions.

Table S1 mbio.00966-25-s0002.xlsx TraDIS and mutation rate assay results.

Table S2 mbio.00966-25-s0003.xlsx TraDIS results in diverse bacteria.

ASM does not own the copyrights to Supplemental Material that may be linked to, or accessed through, an article. The authors have granted ASM a non-exclusive, world-wide license to publish the Supplemental Material files. Please contact the corresponding author directly for reuse.

## References

[1] Swings T, Van den Bergh B, Wuyts S, Oeyen E, Voordeckers K, Verstrepen KJ, Fauvart M, Verstraeten N, Michiels J. 2017. Adaptive tuning of mutation rates allows fast response to lethal stress in Escherichia coli. Elife 6:e22939. doi:10.7554/eLife.22939 28460660 PMC5429094

[2] Matic I. 2019. Mutation rate heterogeneity increases odds of survival in unpredictable environments. Mol Cell 75:421–425. doi:10.1016/j.molcel.2019.06.029 31398322

[3] Matic I. 2013. Edited by D. Mittelman. Stress-induced mutagenesis. Springer, New York, USA.

[4] Sundin GW, Weigand MR. 2007. The microbiology of mutability. FEMS Microbiol Lett 277:11–20. doi:10.1111/j.1574-6968.2007.00901.x 17714481

[5] Wei W, Ho W-C, Behringer MG, Miller SF, Bcharah G, Lynch M. 2022. Rapid evolution of mutation rate and spectrum in response to environmental and population-genetic challenges. Nat Commun 13:4752. doi:10.1038/s41467-022-32353-6 35963846 PMC9376063

[6] Ruis C, Weimann A, Tonkin-Hill G, Pandurangan AP, Matuszewska M, Murray GGR, Lévesque RC, Blundell TL, Floto RA, Parkhill J. 2023. Mutational spectra are associated with bacterial niche. Nat Commun 14:7091. doi:10.1038/s41467-023-42916-w 37925514 PMC10625568

[7] Sniegowski PD, Gerrish PJ, Lenski RE. 1997. Evolution of high mutation rates in experimental populations of E. coli. Nature 387:703–705. doi:10.1038/42701 9192894

[8] Wielgoss S, Barrick JE, Tenaillon O, Wiser MJ, Dittmar WJ, Cruveiller S, Chane-Woon-Ming B, Médigue C, Lenski RE, Schneider D. 2013. Mutation rate dynamics in a bacterial population reflect tension between adaptation and genetic load. Proc Natl Acad Sci USA 110:222–227. doi:10.1073/pnas.1219574110 23248287 PMC3538217

[9] Li G-M. 2008. Mechanisms and functions of DNA mismatch repair. Cell Res 18:85–98. doi:10.1038/cr.2007.115 18157157

[10] Komp Lindgren P, Higgins PG, Seifert H, Cars O. 2016. Prevalence of hypermutators among clinical Acinetobacter baumannii isolates. J Antimicrob Chemother 71:661–665. doi:10.1093/jac/dkv378 26660878 PMC4743697

[11] Li T, Luo D, Ning N, Liu X, Chen F, Zhang L, Bao C, Li Z, Li D, Gu H, Qu F, Yang X, Huang Y, Li B, Wang H. 2023. Acinetobacter baumannii adaptation to the host pH microenvironment is mediated by allelic variation in a single residue of BauA protein. PNAS Nexus 2:gad079. doi:10.1093/pnasnexus/pgad079 PMC10098034 37065616

[12] Marvig RL, Johansen HK, Molin S, Jelsbak L. 2013. Genome analysis of a transmissible lineage of Pseudomonas aeruginosa reveals pathoadaptive mutations and distinct evolutionary paths of hypermutators. PLoS Genet 9:e1003741. doi:10.1371/journal.pgen.1003741 24039595 PMC3764201

[13] Rees VE, Deveson Lucas DS, López-Causapé C, Huang Y, Kotsimbos T, Bulitta JB, Rees MC, Barugahare A, Peleg AY, Nation RL, Oliver A, Boyce JD, Landersdorfer CB. 2019. Characterization of hypermutator Pseudomonas aeruginosa isolates from patients with cystic fibrosis in Australia. Antimicrob Agents Chemother 63:e02538-18. doi:10.1128/AAC.02538-18 30745381 PMC6437500

[14] Gifford DR, Berríos-Caro E, Joerres C, Suñé M, Forsyth JH, Bhattacharyya A, Galla T, Knight CG. 2023. Mutators can drive the evolution of multi-resistance to antibiotics. PLoS Genet 19:e1010791. doi:10.1371/journal.pgen.1010791 37311005 PMC10292718

[15] Taddei F, Radman M, Maynard-Smith J, Toupance B, Gouyon PH, Godelle B. 1997. Role of mutator alleles in adaptive evolution. Nature 387:700–702. doi:10.1038/42696 9192893

[16] Fowler RG, Schaaper RM. 1997. The role of the mutT gene of Escherichia coli in maintaining replication fidelity. FEMS Microbiol Rev 21:43–54. doi:10.1111/j.1574-6976.1997.tb00344.x 9299701

[17] Al Mamun AAM, Lombardo M-J, Shee C, Lisewski AM, Gonzalez C, Lin D, Nehring RB, Saint-Ruf C, Gibson JL, Frisch RL, Lichtarge O, Hastings PJ, Rosenberg SM. 2012. Identity and function of a large gene network underlying mutagenic repair of DNA breaks. Science 338:1344–1348. doi:10.1126/science.1226683 23224554 PMC3782309

[18] Rice LB. 2008. Federal funding for the study of antimicrobial resistance in nosocomial pathogens: no ESKAPE. J Infect Dis 197:1079–1081. doi:10.1086/533452 18419525

[19] Petersen PJ, Jacobus NV, Weiss WJ, Sum PE, Testa RT. 1999. In vitro and in vivo antibacterial activities of a novel glycylcycline, the 9-t-butylglycylamido derivative of minocycline (GAR-936). Antimicrob Agents Chemother 43:738–744. doi:10.1128/AAC.43.4.738 10103174 PMC89200

[20] World Health Organization. 2017. Guidelines for the prevention and control of carbapenem-resistant Enterobacteriaceae, Acinetobacter baumannii and Pseudomonas aeruginosa in health care facilities 29630191

[21] He T, Wang R, Liu D, Walsh TR, Zhang R, Lv Y, Ke Y, Ji Q, Wei R, Liu Z, et al.. 2019. Emergence of plasmid-mediated high-level tigecycline resistance genes in animals and humans. Nat Microbiol 4:1450–1456. doi:10.1038/s41564-019-0445-2 31133751

[22] Hirata T, Saito A, Nishino K, Tamura N, Yamaguchi A. 2004. Effects of efflux transporter genes on susceptibility of Escherichia coli to tigecycline (GAR-936). Antimicrob Agents Chemother 48:2179–2184. doi:10.1128/AAC.48.6.2179-2184.2004 15155219 PMC415592

[23] Peleg AY, Adams J, Paterson DL. 2007. Tigecycline efflux as a mechanism for nonsusceptibility in Acinetobacter baumannii. Antimicrob Agents Chemother 51:2065–2069. doi:10.1128/AAC.01198-06 17420217 PMC1891386

[24] Sun J, Chen C, Cui CY, Zhang Y, Liu X, Cui ZH, Ma XY, Feng Y, Fang LX, Lian XL, Zhang RM, Tang YZ, Zhang KX, Liu HM, Zhuang ZH, Zhou SD, Lv JN, Du H, Huang B, Yu FY, Mathema B, Kreiswirth BN, Liao XP, Chen L, Liu YH. 2019. Plasmid-encoded tet(X) genes that confer high-level tigecycline resistance in Escherichia coli. Nat Microbiol 4:1457–1464. doi:10.1038/s41564-019-0496-4 31235960 PMC6707864

[25] Reid GE, Grim SA, Aldeza CA, Janda WM, Clark NM. 2007. Rapid development of Acinetobacter baumannii resistance to tigecycline. Pharmacotherapy 27:1198–1201. doi:10.1592/phco.27.8.1198 17655518

[26] Cai Y, Wang R, Liang B, Bai N, Liu Y. 2011. Systematic review and meta-analysis of the effectiveness and safety of tigecycline for treatment of infectious disease. Antimicrob Agents Chemother 55:1162–1172. doi:10.1128/AAC.01402-10 21173186 PMC3067123

[27] Hornsey M, Loman N, Wareham DW, Ellington MJ, Pallen MJ, Turton JF, Underwood A, Gaulton T, Thomas CP, Doumith M, Livermore DM, Woodford N. 2011. Whole-genome comparison of two Acinetobacter baumannii isolates from a single patient, where resistance developed during tigecycline therapy. J Antimicrob Chemother 66:1499–1503. doi:10.1093/jac/dkr168 21565804

[28] Hammerstrom TG, Beabout K, Clements TP, Saxer G, Shamoo Y. 2015. Acinetobacter baumannii repeatedly evolves a hypermutator phenotype in response to tigecycline that effectively surveys evolutionary trajectories to resistance. PLoS ONE 10:e0140489. doi:10.1371/journal.pone.0140489 26488727 PMC4619398

[29] Cain AK, Barquist L, Goodman AL, Paulsen IT, Parkhill J, van Opijnen T. 2020. A decade of advances in transposon-insertion sequencing. Nat Rev Genet 21:526–540. doi:10.1038/s41576-020-0244-x 32533119 PMC7291929

[30] Boinett CJ, Cain AK, Hawkey J, Do Hoang NT, Khanh NNT, Thanh DP, Dordel J, Campbell JI, Lan NPH, Mayho M, Langridge GC, Hadfield J, Chau NVV, Thwaites GE, Parkhill J, Thomson NR, Holt KE, Baker S. 2019. Clinical and laboratory-induced colistin-resistance mechanisms in Acinetobacter baumannii. Microb Genom 5:e000246. doi:10.1099/mgen.0.000246 30720421 PMC6421349

[31] Rosenfeld N, Bouchier C, Courvalin P, Périchon B. 2012. Expression of the resistance-nodulation-cell division pump AdeIJK in Acinetobacter baumannii is regulated by AdeN, a TetR-type regulator. Antimicrob Agents Chemother 56:2504–2510. doi:10.1128/AAC.06422-11 22371895 PMC3346617

[32] Gerson S, Nowak J, Zander E, Ertel J, Wen Y, Krut O, Seifert H, Higgins PG. 2018. Diversity of mutations in regulatory genes of resistance-nodulation-cell division efflux pumps in association with tigecycline resistance in Acinetobacter baumannii. J Antimicrob Chemother 73:1501–1508. doi:10.1093/jac/dky083 29554339

[33] Goh KGK, Phan MD, Forde BM, Chong TM, Yin WF, Chan KG, Ulett GC, Sweet MJ, Beatson SA, Schembri MA. 2017. Genome-wide discovery of genes required for capsule production by uropathogenic Escherichia coli. MBio 8:1–16. doi:10.1128/mBio.01558-17 PMC5654933 29066548

[34] Dorman MJ, Feltwell T, Goulding DA, Parkhill J, Short FL. 2018. The capsule regulatory network of Klebsiella pneumoniae defined by density-TraDISort. MBio 9:e01863-18. doi:10.1128/mBio.01863-18 30459193 PMC6247091

[35] Domingues S, Rosário N, Cândido Â, Neto D, Nielsen KM, Da Silva GJ. 2019. Competence for natural transformation is common among clinical strains of resistant Acinetobacter spp. Microorganisms 7:30. doi:10.3390/microorganisms7020030 30682786 PMC6406254

[36] Maki H, Sekiguchi M. 1992. MutT protein specifically hydrolyses a potent mutagenic substrate for DNA synthesis. Nature 355:273–275. doi:10.1038/355273a0 1309939

[37] He F, Shi Q, Fu Y, Xu J, Yu Y, Du X. 2018. Tigecycline resistance caused by rpsJ evolution in a 59-year-old male patient infected with KPC-producing Klebsiella pneumoniae during tigecycline treatment. Infect Genet Evol 66:188–191. doi:10.1016/j.meegid.2018.09.025 30268919

[38] Garibyan L, Huang T, Kim M, Wolff E, Nguyen A, Nguyen T, Diep A, Hu K, Iverson A, Yang H, Miller JH. 2003. Use of the rpoB gene to determine the specificity of base substitution mutations on the Escherichia coli chromosome. DNA Repair (Amst) 2:593–608. doi:10.1016/s1568-7864(03)00024-7 12713816

[39] Cain AK, Boinett CJ, Barquist L, Dordel J, Fookes M, Mayho M, Ellington MJ, Goulding D, Pickard D, Wick RR, Holt KE, Parkhill J, Thomson NR. 2018. Morphological, genomic and transcriptomic responses of Klebsiella pneumoniae to the last-line antibiotic colistin. Sci Rep 8:9868. doi:10.1038/s41598-018-28199-y 29959380 PMC6026146

[40] A Ghomi F, Jung JJ, Langridge GC, Cain AK, Boinett CJ, Abd El Ghany M, Pickard DJ, Kingsley RA, Thomson NR, Parkhill J, Gardner PP, Barquist L. 2024. High-throughput transposon mutagenesis in the family Enterobacteriaceae reveals core essential genes and rapid turnover of essentiality. mBio 15:e0179824. doi:10.1128/mbio.01798-24 39207104 PMC11481867

[41] Sharp C, Boinett C, Cain A, Housden NG, Kumar S, Turner K, Parkhill J, Kleanthous C. 2019. O-antigen-dependent colicin insensitivity of uropathogenic Escherichia coli*.* J Bacteriol 201:e00545-18. doi:10.1128/JB.00545-18 30510143 PMC6351738

[42] Barquist L, Mayho M, Cummins C, Cain AK, Boinett CJ, Page AJ, Langridge GC, Quail MA, Keane JA, Parkhill J. 2016. The TraDIS toolkit: sequencing and analysis for dense transposon mutant libraries. Bioinformatics 32:1109–1111. doi:10.1093/bioinformatics/btw022 26794317 PMC4896371

[43] Khan A, Mathelier A. 2017. Intervene: a tool for intersection and visualization of multiple gene or genomic region sets. BMC Bioinformatics 18:287. doi:10.1186/s12859-017-1708-7 28569135 PMC5452382

[44] Gallagher LA, Ramage E, Weiss EJ, Radey M, Hayden HS, Held KG, Huse HK, Zurawski DV, Brittnacher MJ, Manoil C. 2015. Resources for genetic and genomic analysis of emerging pathogen Acinetobacter baumannii. J Bacteriol 197:2027–2035. doi:10.1128/JB.00131-15 25845845 PMC4438207

[45] Luria SE, Delbrück M. 1943. Mutations of bacteria from virus sensitivity to virus resistance. Genetics 28:491–511. doi:10.1093/genetics/28.6.491 17247100 PMC1209226

[46] Sarkar S, Ma WT, Sandri GH. 1992. On fluctuation analysis: a new, simple and efficient method for computing the expected number of mutants. Genetica 85:173–179. doi:10.1007/BF00120324 1624139

[47] Hall BM, Ma C-X, Liang P, Singh KK. 2009. Fluctuation analysis CalculatOR: a web tool for the determination of mutation rate using Luria-Delbruck fluctuation analysis. Bioinformatics 25:1564–1565. doi:10.1093/bioinformatics/btp253 19369502 PMC2687991

