# Supplementary material for: Transposon insertion sequencing reveals novel hypermutator genes in Acinetobacter baumannii
Source: mBio. 2025 Jun 27;16(8):e00966-25. doi: 10.1128/mbio.00966-25 (PMC12345246; doi:10.1128/mbio.00966-25)
Supplement: Supplemental material — Supplemental figures and table captions. [file mbio.00966-25-s0001.docx]

**Supplemental material**

**Supplemental Figure 1**

**Supplemental Figure 2**

**Table S1** Full *A. baumannii* tigecycline TraDIS results (excel file)

**Table S2** Analysis of TraDIS datasets from four species for hypermutator selection (excel file)


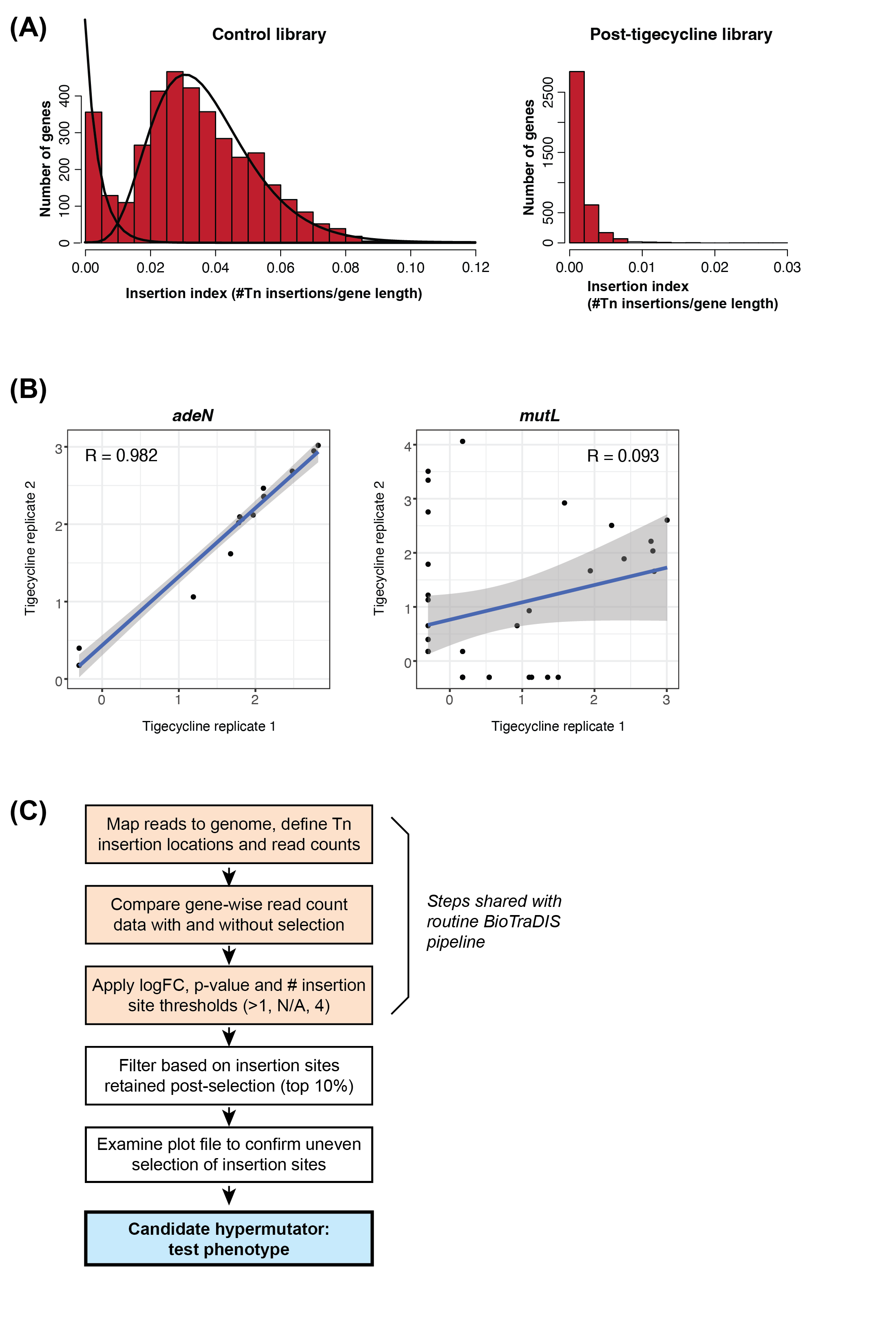


**Figure S1**. (A) Insertion index histogram showing collapse in transposon library diversity during tigecycline selection. While the control library shows a range of insertion index values with clear peaks for non-essential and essential genes, the majority of genes have a very low insertion index following tigecycline treatment (right) and essential and non-essential genes can no longer be distinguished. (B) Correlation between log10-transformed read counts at each mapped insertion site following tigecycline treatment for a gene where inactivation increases fitness directly hit (*adeN*, left), and a gene where inactivation indirectly increases fitness (*mutL*, right). (C) Workflow for identifying putative hypermutator genes in TIS datasets.

**
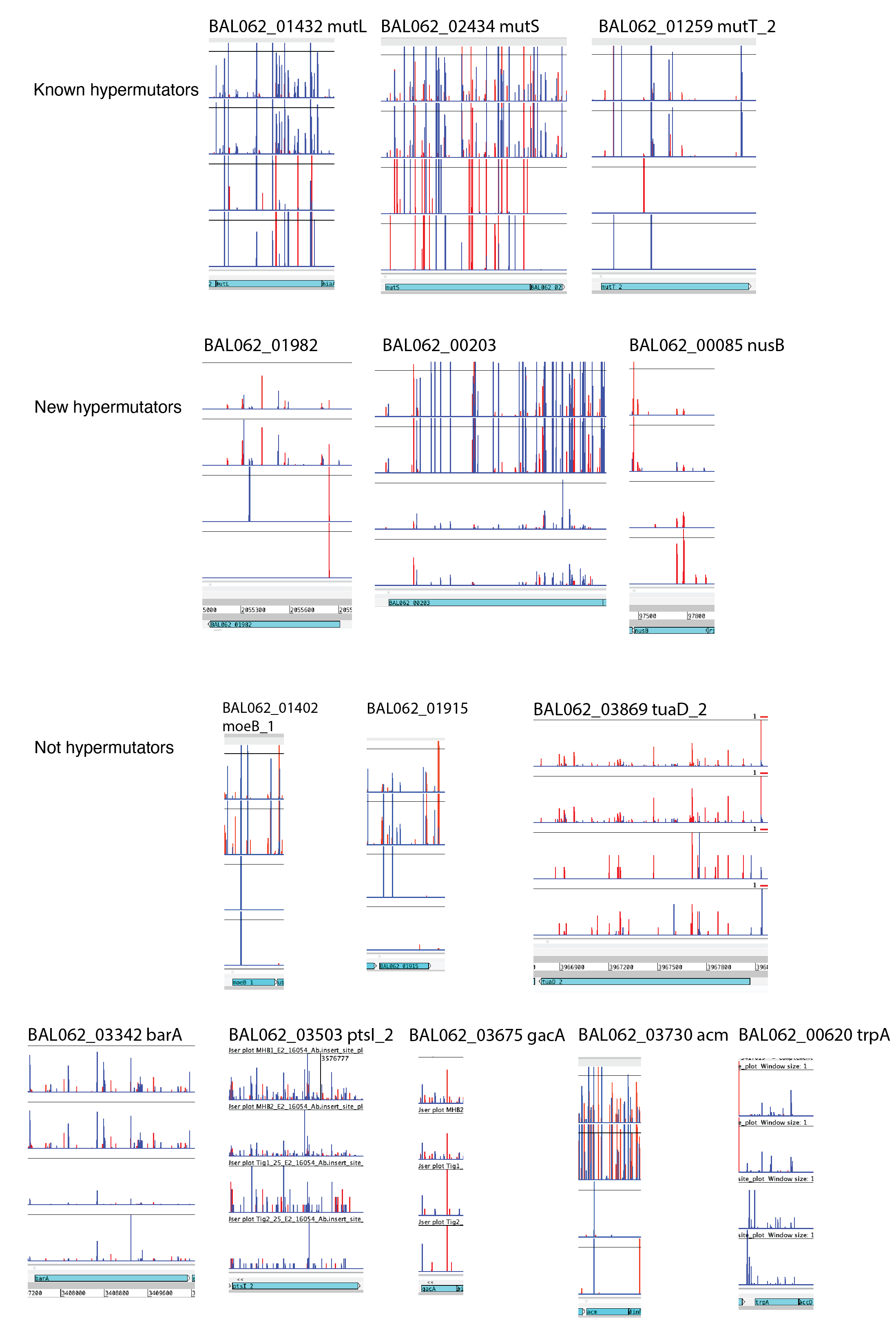
Figure S2**. Insertion site plots for genes tested for hypermutator phenotypes. Individual transposon insertion locations are indicated by vertical lines, with height proportional to the number of reads mapped. Red and blue indicate the orientation of transposon insertions. Each image shows the results from the control condition (top two plots), and post tigecycline treatment (bottom two plots). Profiles are shown for the genes which were known hypermutators, genes shown in this study to have a previously unknown hypermutator phenotype, and genes which were tested and found not to have elevated mutation rates. Note that the non-hypermutator genes include two (*moeB_1* and *acm*) with the secondary mutation – not hypermutator profile (see Figure 1) which were included as negative controls.
